# Integrative profiling of transcriptome and metabolome of skeletal muscle after endurance exercise in a high-fat diet

**DOI:** 10.1016/j.clinsp.2026.101009

**Published:** 2026-05-28

**Authors:** Ning Jiang, Yu Song, Zhe Wang, Yumo Dong, Qian Wang, Huaduo Wu, Xin Gao, Yaning Song, Yunlong Cui

**Affiliations:** a Tianjin Key Laboratory of Exercise Physiology and Sports Medicine, Institute of Sport, Exercise & Health, Tianjin University of Sport, Tianjin, China; b Department of Basic Teaching of Military Common Subjects, Logistics University of Chinese People’s Armed Police Force, Tianjin, China; c Department of Hepatobiliary Surgery, Tianjin Medical University Cancer Institute & Hospital, Tianjin, China

**Keywords:** Endurance exercise, Obesity, Skeletal muscle transcriptome, Skeletal muscle metabolome

## Abstract

**Objective:**

Regular exercise reduces the risk of obesity, but the impact of endurance exercise on skeletal muscle metabolic processes remains incompletely understood. This study investigated the effects of endurance exercise on the transcriptome and metabolome of skeletal muscle in obese mice.

**Method:**

Transcriptomic and metabolomic profiling of skeletal muscle samples was performed across experimental groups to identify differentially expressed genes and differential metabolites. Functional enrichment of DEGs and metabolites was analyzed using Gene Ontology (GO) and Kyoto Encyclopedia of Genes and Genomes (KEGG) pathway analyses. Integrated pathway-level analysis was conducted using iPath 3.0.

**Results:**

Integrated transcriptomic and metabolomic analysis indicated that endurance exercise altered lipid metabolism, amino acid metabolism, and nucleotide metabolism pathways in the skeletal muscle of obese mice. Specifically, endurance exercise downregulated the expression of lipid metabolism-related gene *Acot2*, amino acid metabolism-related gene *Nos2*, and carbohydrate metabolism-related genes *Itpkb* and *Inpp4b*. Furthermore, endurance exercise decreased the levels of palmitic acid, arachidonate, phosphatidylcholine, acetylcholine, hypoxanthine, urea, and acetyl-CoA.

**Conclusions:**

Endurance exercise positively impacts skeletal muscle metabolic health in obese mice through coordinated regulation of multiple metabolic pathways and reduction of specific metabolites

## Highlights

• Exercise regulates lipid and nucleotide metabolism genes in obese muscle.
• Exercise alters lipid, amino acid, and nucleotide pathways in obese mice.
• Exercise reduces key metabolites and gene expression to improve metabolic health.

## Introduction

Obesity is a chronic metabolic condition characterized by an excessive accumulation of body fat that poses serious health risks. Obesity is associated with numerous chronic diseases, such as cardiovascular disease^1^, diabetes mellitus^2^, and certain types of cancer^3^ and musculoskeletal disorders.^4^ It is estimated that in 2019, excess body mass index caused 5-million deaths from non-communicable diseases such as diabetes and cardiovascular disease.^5^ Currently, the commonly prescribed obesity treatment drugs include orlistat^6^, phentermine/topiramate^7^, and naltrexone/bupropion.^8^ While effective for promoting weight loss, these medications come with a variety of side effects, such as flatulence, and abdominal discomfort^9^, constipation, insomnia, nausea, headache and increased heart rate^10^^,^^11^ that can impact patients' comfort and daily life.

Skeletal muscle is the largest metabolic and endocrine organ of the human body and plays a vital role in maintaining the normal function and metabolic balance of the musculoskeletal system.^12^ Obesity promotes inflammation of adipose tissue and increases lipolysis, which will lead to ectopic fat accumulation in skeletal muscles, leading to impairment of skeletal muscle function.^13^ Previous research shows significant differences in glucose and lipid metabolism between obese and lean people.^14^ In individuals with severe obesity, alterations in the glucose metabolism of skeletal muscle have been noted, including diminished insulin-mediated glucose uptake, decreased oxidative metabolism, and an increase in lactate production.^15^^,^^16^ In individuals with obesity, the level of triacylglycerol is increased, and lipid antioxidant capacity is decreased in skeletal muscle.^17^^,^^18^ Thus, finding effective strategies to improve skeletal muscle metabolic disorders and functional impairment is crucial to promote healthy activities in individuals with obesity.

Physical activity and exercise are well-established techniques to prevent and treat individuals with obesity.^19^ A systematic literature review conducted that endurance exercise at moderate intensity is particularly beneficial for reducing body weight, total fat, and visceral fat, as well as improving blood pressure.^20^ Moreover, endurance exercise improves obesity-related metabolic abnormalities.^21^ Endurance training has demonstrated the ability to simultaneously enhance lipid oxidation capacity and increase the lipolytic protein levels in the skeletal muscle of obese individuals.^22^ Endurance exercise downregulates the acetyl-CoA production pathway in the liver of mice fed a high-energy diet and increases mitochondrial mass and mitochondrial oxidative capacity in skeletal muscle.^23^ However, endurance exercise performance requires complex multifactorial processes, the mechanisms of which improve skeletal muscle metabolic processes are not fully understood. In addition, most previous studies have examined gene expression or metabolic alterations in isolation, which limits their understanding of the complex regulatory networks involved. Therefore, integrating transcriptomic and metabolomic analyses may provide a more comprehensive view of the molecular and metabolic adaptations in skeletal muscle under obese conditions. Researching the skeletal muscle transcriptome and metabolome in obesity is essential for unraveling the molecular changes that occur in muscle tissue, which may inspire therapeutic approaches to ameliorate the effects of obesity on muscle health.

## Material and methods

### Animals and diets

Forty-eight male 7-week-old C57BL/6 mice (body weight: 20 ± 1 g) were obtained from Beijing Vitong Lihua Laboratory Animal Technology Co., Ltd (Beijing, China) and fed a regular chow diet to adapt to the environment. This study was carried out in accordance with the principles of the Basel Declaration (https://animalresearchtomorrow.org/en). All animal experiments were performed in compliance with the ARRIVE guidelines and were approved by the Animal Experiment Ethical Review Committee of Tianjin University of Sport’s Medical Ethics Committee (TJUS2023–010). Animal experiments were carried out with the approval of the institutional Animal Care Committee in accordance with Canadian Council on Animal Care (CCAC) guidelines.

All mice were housed under controlled conditions (12 h light-dark cycle, 22∼25 °C) with free access to distilled water and food. After 1-week, 48 mice were randomly divided into two groups: Normal Chow diet (NC, *n* = 16) and High-Fat Diet (HFD, *n* = 32) groups. The mice in the NC group were fed a normal chow diet, which included ≤ 10.0% moisture, ≥ 18% crude protein, ≥ 4.0% crude fat, ≤ 5.0% crude fiber, ≤8.0% crude ash, and 1.0%–1.8% calcium, along with 0.6%–1.2% phosphorus (Beijing Huafukang Bioscience Co., Ltd). In contrast, the HFD group was fed an HFD consisting of 20% kcal from protein, 60% kcal from fat, and 20% kcal from carbohydrates, also sourced from Beijing Huafukang Bioscience Co., Ltd, Beijing, China, for a duration of 12-weeks. After a duration of 12-weeks, it was observed that the body weights of 16 mice in the HFD group exceeded 20% of the average body weights of the mice in the control group. Subsequently, the mice in the Normal Control (NC) group were randomly divided into two distinct categories: the NC control group (NC, *n* = 3) and the NC exercise group (NE, *n* = 3). Similarly, the HFD mice were also allocated into two groups: the HFD control group (HFD, *n* = 3) and the HFD exercise group (HFE, *n* = 3). It is important to note that the mice belonging to both the NC and HFD groups did not undergo any endurance exercise training. In contrast, the mice categorized into the NE and HFE groups participated in an 8-week regimen of endurance exercise training conducted on a treadmill. Body weight and food intake were measured and recorded every week. After experiment completion, mice were euthanized by cervical dislocation.

### Endurance training

Mice in the NE and HFE groups underwent a one-week pre-exercise training session, during which the training duration and speed were gradually increased from 20 min at 8 m/min to 50 min at 10 m/min. Following this, the mice exercised on a treadmill for 60 min at an intensity regimen consisting of 10 m/min for 10 min, 12 m/min for 40 min, and 10 m/min for an additional 10 min, all at a 0% grade for a duration of 1 to 4-weeks. During weeks 5 to 8, the exercise intensity was increased to 10 m/min for 10 min, 14 m/min for 40 min, and 10 m/min for 10 min, maintaining the same slope, with a total of 60 min per session and 5 sessions per week, while allowing the mice to rest on Saturdays. Body weight and food intake of the mice were recorded weekly throughout the exercise intervention. At the conclusion of the intervention, one mouse from each of the NE and HFE groups unfortunately died due to an accident.

### RNA extraction and skeletal muscle transcriptome analysis

Total RNA extraction was performed utilizing a Trizol reagent kit (Invitrogen, Carlsbad, CA, USA), following the guidelines provided by the manufacturer. RNA quality was assessed using an Agilent 2100 Bioanalyzer (Agilent Technologies, Palo Alto, CA, USA) and RNase-free agarose gel electrophoresis. Only high-quality RNA samples (RNA integrity number, RIN ≥ 7.0) were used for library construction. Following the extraction of total RNA, eukaryotic mRNA was enriched using Oligo (dT). Subsequently, the enriched mRNA was fragmented into smaller segments using the fragmentation buffer and then reverse-transcribed into cDNA utilizing the NEBNext Ultra RNA Library Prep Kit for Illumina (NEB#7530, New England Biolabs, Ipswich, MA, USA). The end repair of the isolated double-stranded cDNA fragments was performed, followed by the addition of an A base, before ligating them to the Illumina sequencing adaptors. The purification of the ligation reaction was carried out using AMPure XP Beads at a ratio of 1.0X. To amplify the DNA quantity, the Polymerase Chain Reaction (PCR) technique was employed. The resulting cDNA library was sequenced by Gene Denovo Biotechnology Co., based in Guangzhou, China, utilizing the Illumina Novaseq6000 platform.

Raw sequencing reads were filtered using fastp (version 0.18.0) to obtain high-quality clean reads. The filtering parameters were as follows: 1) Removal of reads containing adapters; 2) Removal of reads containing more than 10% unknown Nucleotides (N); and 3) Removal of low-quality reads containing more than 50% of bases with a *Q*-value ≤ 20. Clean reads were then aligned to the ribosomal RNA (rRNA) database using Bowtie2 (version 2.2.8), and rRNA-mapped reads were removed. The remaining clean reads were mapped to the mouse reference genome using HISAT2 (version 2.2.4). For each transcription region, gene expression abundance was calculated and normalized to Fragments per Kilobase of transcript per million Mapped reads (FPKM) using RSEM software to eliminate the influence of gene length and sequencing depth on gene expression calculations.

### Untargeted skeletal muscle metabolome analysis

The extraction of metabolites from skeletal muscle samples was conducted using a methanol/acetonitrile solution in a 1:1 ratio (v/v). The mixture was subjected to centrifugation at 14,000 g for 15 min at 4 °C. Following this, the supernatant was concentrated using a vacuum centrifuge. The resulting samples were then reconstituted in 100 μL of an acetonitrile/water solvent mixture (1:1, v/v) for LC-MS analysis. To monitor instrument stability and data reliability, Quality Control (QC) samples were prepared by pooling equal aliquots of each sample and analyzed at regular intervals throughout the sequence.

The LC-MS/MS analysis was carried out by Shanghai Applied Protein Technology Co., Ltd., utilizing a UHPLC system (1290 Infinity LC, Agilent Technologies) coupled with a quadrupole time-of-flight (AB Sciex TripleTOF 6600). Chromatographic separation was performed on an ACQUITY UPLC BEH Amide column (2.1 × 100 mm, 1.7 μm) at 25 °C. The mobile phase consisted of A (25 mM ammonium acetate and 25 mM ammonium hydroxide in water) and B (acetonitrile). The gradient elution program was as follows: 0–0.5 min, 95% B; 0.5–7 min, 95% to 65% B; 7–8 min, 65% to 40% B; 8–9 min, 40% B; 9–9.1 min, 40% to 95% B; 9.1–12 min, 95% B. The flow rate was 0.5 mL/min, and the injection volume was 2 μL. Samples were maintained at 4 °C in the autosampler throughout the analysis. Mass spectrometry was performed in both positive and negative ion modes with the following ESI source parameters: Ion Source Gas1: 60, Ion Source Gas2: 60, Curtain Gas: 30, source temperature: 600 °C, IonSpray Voltage Floating: ± 5500 V. For the TOF MS scan, the *m/z* range was 60–1000 Da with an accumulation time of 0.20 s/spectra. For product ion scan, the *m/z* range was 25–1000 Da with an accumulation time of 0.05 s/spectra. Data-dependent Acquisition (IDA) was used to acquire MS/MS spectra in high sensitivity mode.

Raw MS data were converted to mzXML format using ProteoWizard and processed using XCMS software for peak alignment, retention time correction, and peak area extraction. The XCMS parameters were set as follows: for peak picking, centWave *m/z* = 10 ppm, peakwidth = c(10, 60), prefilter = c(10, 100); for peak grouping, bw = 5, mzwid = 0.025, minfrac = 0.5. Metabolites with > 50% missing values in any group were removed, and remaining missing values were imputed using the K-Nearest Neighbor (KNN) method. Data were normalized by total peak area. Metabolite identification was performed by comparing accurate *m/z* values (< 10 ppm) and MS/MS spectra with an in-house database established with authentic standards.

### Identification of differentially expressed genes (DEGs)

**Fig. 1:**
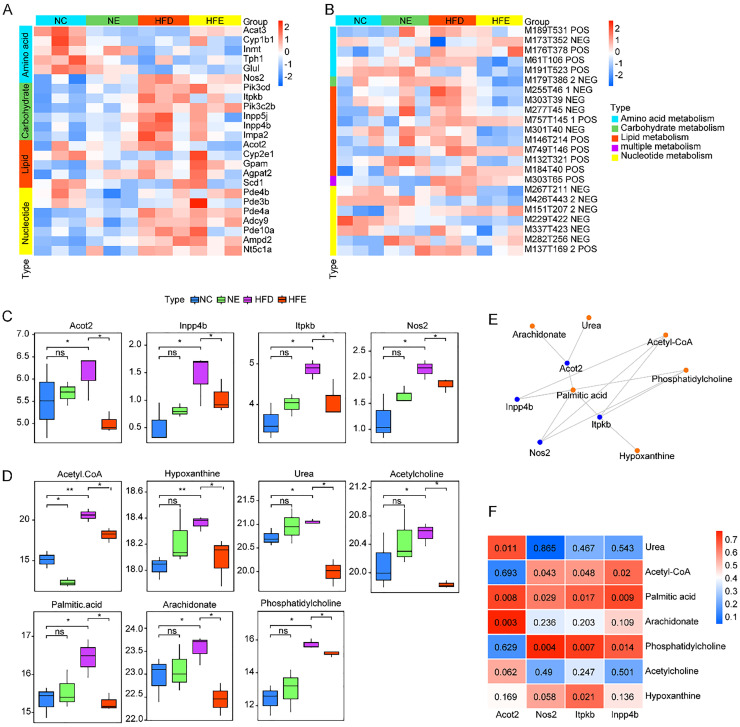
Integrated transcriptome and metabolome analysis of the key metabolic pathway. Heatmap of expression levels of key genes (A) and abundance of key metabolites (B). (C) The mRNA expression levels of key metabolism-related genes in different groups. (D) The levels of key metabolites in different groups. (E) Key genes and key metabolites network diagram. (F) Heatmap of correlation between key genes and key metabolites.

The R language “FactoMineR” function package (version 2.11) was used to establish the Principal Component Analysis (PCA) model, and the contributions of the first two axes were extracted as reference values for differences between and within groups. Based on the DESeq2 (version 1.42.1)^24^ function package in the R language, the “estimateSizeFactors” function was used to standardize the number of counts to obtain the basemean value. The DESeq function was used to perform significance testing and calculate the p-value and Fold-Change value of the difference comparison. To control the false positive phenomenon caused by multiple comparisons, the authors employed the Benjamini-Hochberg method to calculate the False Discovery Rate (FDR) and performed a correction for multiple hypothesis testing on the p-values. The DEGs were identified using a threshold of |log2 FC| > 1 and FDR < 0.05. For visualization of gene expression levels across samples (e.g., in Fig. 1A and C), Fragments Per Kilobase of transcript per Million mapped reads (FPKM) values were calculated to normalize for gene length and sequencing depth.

### Identification of differential metabolites

PCA and Orthogonal Partial Least Squares Discriminant Analysis (OPLS-DA) were used to analyze metabolic changes between different groups. The R language “ropls” function package (version 1.34.0) was used to establish the PCA model and OPLS-DA model, and calculate the co-correlation coefficients of the main components and metabolites, the correlation coefficients of the main components and metabolites, and construct the S-plot model. The OPLS-DA model was validated using cross-validation and permutation testing (200 permutations). Model predictive ability was assessed using *Q*^2^ values, with *Q*^2^ > 0.4 considered acceptable. The differential metabolites in different groups were identified using VIP ≥ 1 and p-value < 0.05.

### Functional enrichment analysis

The functional information of DEGs was explored using Gene Ontology (GO) and Kyoto Encyclopedia of Genes and Genomes (KEGG) enrichment analysis. The highly enriched pathways were identified via p < 0.05 and q < 0.05. For the analysis of metabolite abundance data, Kobas software (version 2.1.1) was utilized to calculate the number of metabolites annotated to various pathways. The differentially enriched metabolite pathways were screened using a p-value < 0.05.

Additionally, integrated transcriptomic and metabolomic analyses were performed using iPath 3.0 to examine significantly altered metabolic pathways.

### Statistical analysis

The Wilcoxon rank sum test was used to compare differences in genes or metabolites between groups. Use the R language to perform Pearson correlation analysis; p < 0.05 indicates that the difference is statistically significant. All statistical analyses were performed using R software version 4.3.3.

## Results

### Effects of endurance exercise on skeletal muscle transcriptome

The previous research showed that endurance exercise can reduce body weight and body fat index in mice fed an HFD.^25^ Studies have shown that obesity promotes inflammation of adipose tissue, increases lipolysis, leads to ectopic fat accumulation in skeletal muscles, and ultimately damages the function of skeletal muscle.^13^ Thus, in this study, the authors analyzed the effects of resistance exercise on the skeletal muscle transcriptome and metabolism.

**Fig. 2:**
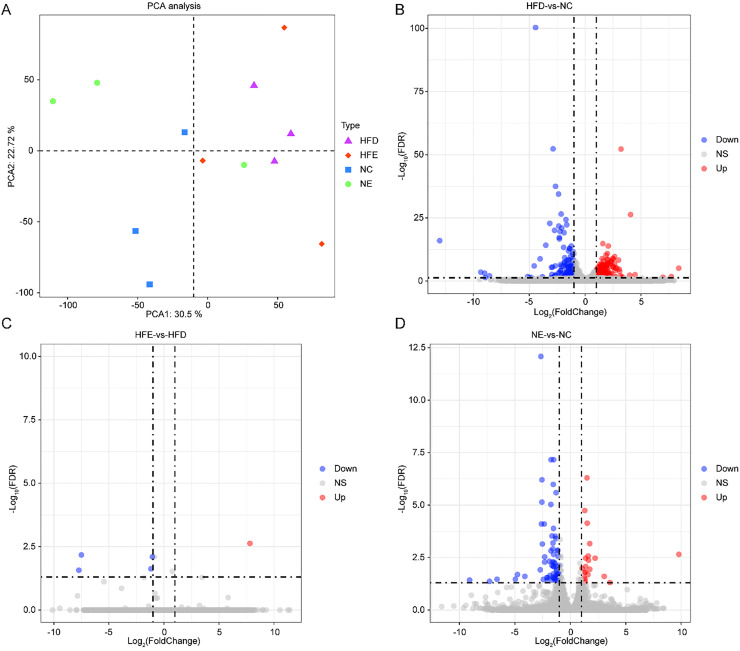
Effects of endurance exercise on skeletal muscle transcriptome. (A) Principal Component Analysis (PCA) plot. The differentially expressed genes between HFD and NC groups (B), HFE and HFD groups (C), and NC and NE groups (D). HFD, HFE, NC, and NE, with three mice in each group.

RNA sequencing analysis was performed on skeletal muscle from HFD, HFE, NC, and NE groups. Endurance exercise separated HFD groups from NC groups (Fig. 2A). There was a significant difference between the HFD group and the NC group (Fig. S1A). All samples in both the NC and NE groups were distinctly separated (Fig. S1B). However, the HFE group showed slight changes compared with the HFD group (Fig. S1C). Differential expression analysis showed that compared to the NC group, 414 DEGs were identified in the HFD group (Fig. 2B, Table S1, |log2 FC| > 1 and FDR < 0.05). Relative to HFD, only 5 DEGs were identified in the HFE group (Fig. 2C, Table S1, |log2 FC| > 1 and FDR < 0.05). Compared to the NC group, the NE group exhibited 81 DEGs (Fig. 2D, Table S1, |log2 FC| > 1 and FDR < 0.05).

### Enrichment analysis of DEGs

**Fig. 3:**
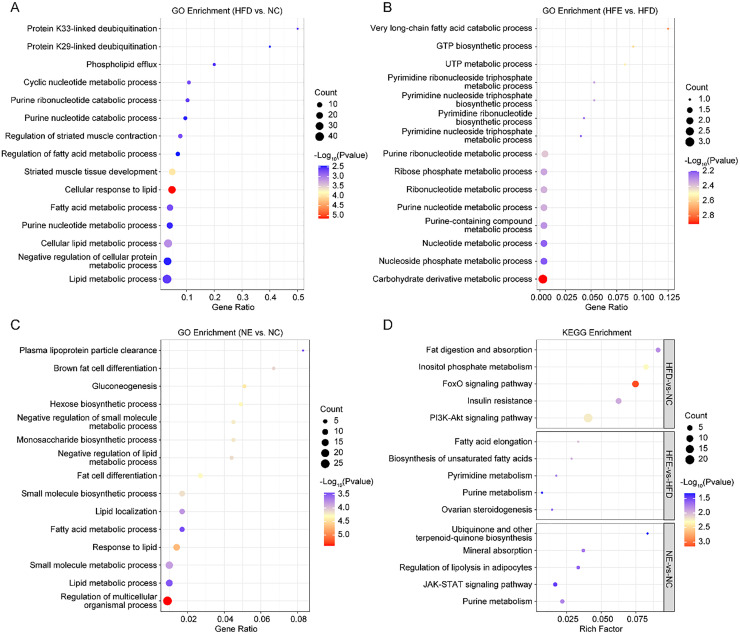
Enrichment analysis of DEGs. The top 10 significantly enriched GO terms in HFD vs. NC (A), HFE vs. HFD (B), and NC vs. NE (C). The top 5 significantly enriched KEGG pathways in HFD vs. NC, HFE vs. HFD, and NC vs. NE.

The potential function information of DEGs induced by diet factors and endurance exercise was characterized using GO and KEGG enrichment analysis. GO analysis showed that DEGs (HFD vs. NC) were significantly enriched in 779 biological processes, such as lipid metabolic and fatty acid metabolic processes (Fig. 3A, Table S2). Compared to the HFD group, the carbohydrate derivative metabolic and nucleotide metabolic process enriched in the HFE group (Fig. 3B, Table S2). Regulation of multicellular organismal process, lipid metabolic process, small molecule metabolic process, and response to lipid and fat cell differentiation were mainly enriched in NC vs. NE (Fig. 3C, Table S2). It is worth noting that the small molecule metabolic process was enriched in all three comparisons (Fig. S2).

KEGG showed that DEGs (HFD vs. NC) were significantly enriched in 56 signaling pathways, such as fat digestion and absorption, inositol phosphate metabolism, and the FoxO signaling pathway (Fig. 3D, Table S2). The DEGs between HFE and HFD were mainly enriched in fatty acid elongation and purine metabolism signaling pathways (Fig. 3D, Table S2). Moreover, purine metabolism, JAK-STAT signaling pathway, and regulation of lipolysis in adipocytes were mainly enriched in NE vs. NC (Fig. 3D, Table S2).

### Effects of endurance exercise on skeletal muscle metabolic profiles

**Fig. 4:**
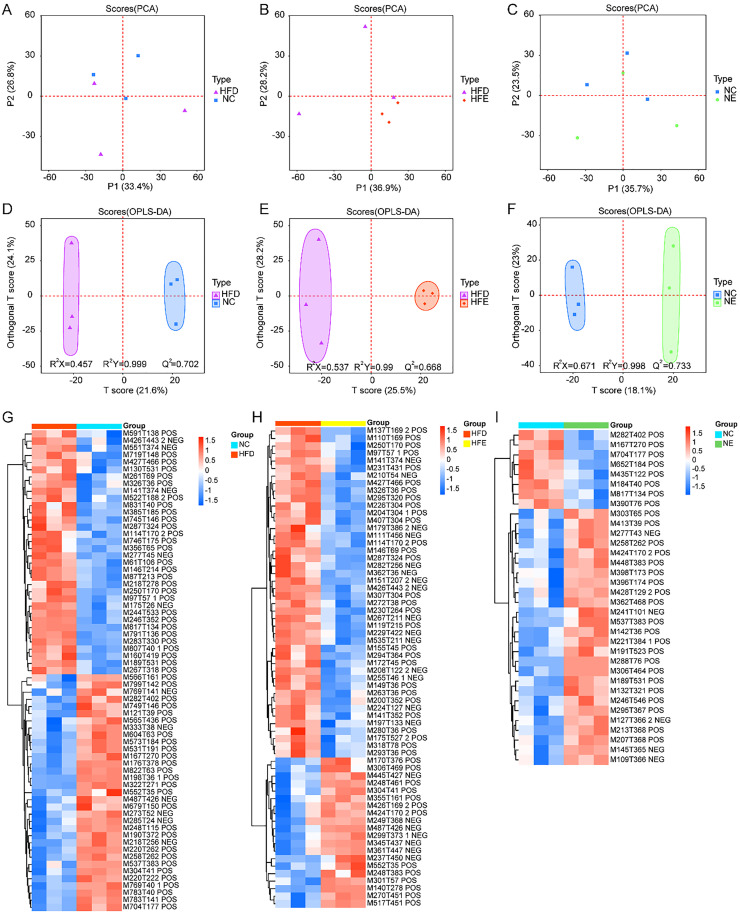
Effects of endurance exercise on skeletal muscle metabolic profiles. PCA plot of skeletal muscle metabolic profiles between HFD and NC (A), HFE and HFD (B), NE and NC groups (C). OPLS-DA score plot in HFD vs. NC (D), HFE vs. HFD (E), and NC vs. NE (F). Differential metabolites between HFD and NC group (G), HFE and HED groups (H), and NC and NE groups (I).

UPLC-MS/MS was used to compare metabolic changes between HFD and NC, HFE and HFD, and NC and NE. A total of 2442 metabolites were identified, including 1055 anion mode metabolites and 1387 cation mode metabolites. PCA showed that there were clear differences between HFD and NC (Fig. 4A), HFE and HFD (Fig. 4B), NE and NC groups (Fig. 4C). Additionally, OPLS-DA was performed between the NCD and HFD groups, HFD and HGH groups, and NCD and NGH groups, respectively. The predictive ability (*Q*^2^) value of HFD vs. NC (Fig. 4D), HFE vs. HFD (Fig. 4E), and NC vs. NE (Fig. 4F) was 0.702, 0.688, and 0.733, respectively, indicating the reliability of the proposed models. The loadings S-plot showed that there were significant differences in metabolites between HFD and NC groups, HFE and HFD groups, and NC and NE groups, respectively (Fig. S3). A total of 67 differential metabolites were identified between the HFD and NC groups (Fig. 4G). There were 64 differential metabolites between HFE and HED groups (Fig. 4H), and 34 differential metabolites between NC and NE groups (Fig. 4I) (VIP ≥ 1 and p-value < 0.05).

**Fig. 5:**
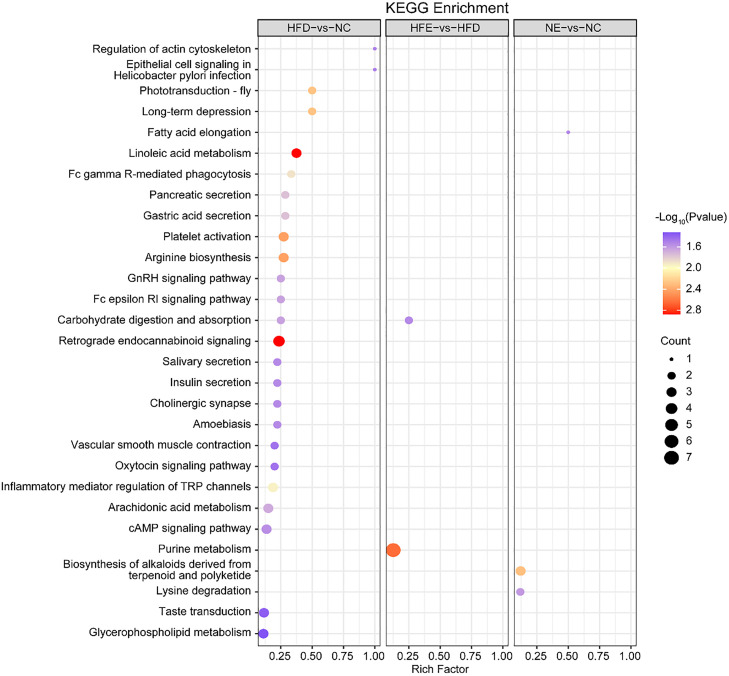
Bubble diagram of enriched KEGG pathways of differential metabolites. Size of dots represents number of metabolites in each KEGG pathway.

KEGG results showed that differential metabolites between HFD and NC groups were significantly enriched in 25 signaling pathways, including glycerophospholipid metabolism, retrograde endocannabinoid signaling, and carbohydrate digestion and absorption pathway (Fig. 5, Table S3). The regulatory metabolites of HFE and HFD mainly affect purine metabolism and carbohydrate digestion and absorption pathways (Fig. 5, Table S3). Biosynthesis of alkaloids derived from terpenoid and polyketide, lysine degradation, and fatty acid elongation pathways were mainly enriched in NC vs. NE (Fig. 5, Table S3). Notably, the carbohydrate digestion and absorption pathway was enriched in HFD vs. NC and HFE vs. HFD (Fig. 5).

### Integrated analysis of transcriptome and metabolome

**Fig. 6:**
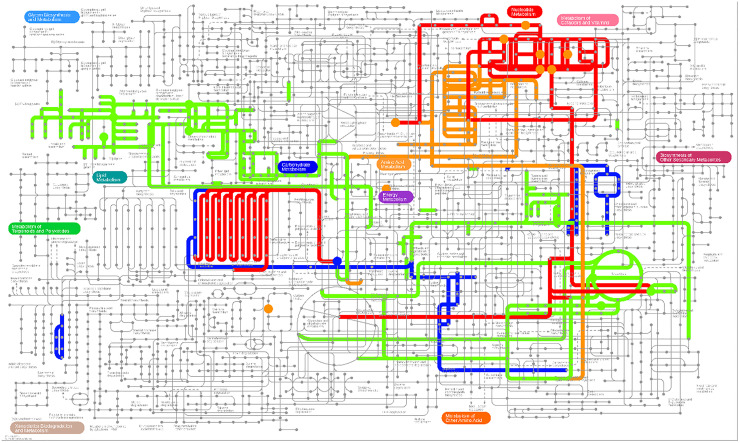
iPath analysis of transcriptome and metabolome results. Dots represent differential metabolites, lines represent metabolic pathways enriched in differential genes and metabolites, blue represents differential pathways and metabolites in the NE vs. NC group, green represents differential pathways and metabolites in HFD vs. NC, and orange represents differential pathways and metabolites in the HFE vs. HFD group. The red color represents the overlapping results of the NE vs. NC group and the HFE vs. HFD group.

Furthermore, the authors further explored altered metabolic pathway response to endurance exercise via integrating differential metabolites and DEGs for in-depth analysis in iPath 3.0 (Fig. 6). Altered metabolic pathways in comparisons of HFE vs. NC, HFE vs. HFD, and NC vs. NE were mainly enriched in lipid metabolism (fatty acid elongation, biosynthesis of unsaturated fatty acids, glycerophospholipid metabolism, arachidonic acid metabolism, linoleic acid metabolism), amino acid metabolism (arginine biosynthesis, lysine degradation, tryptophan metabolism), and nucleotide metabolism (Purine metabolism) pathways (Fig. 6). Thus, the authors hypothesized that endurance exercise might alleviate HFD-induced obesity via regulating lipid metabolism, amino acid metabolism, and nucleotide metabolism pathways. Thus, the authors, analyzed the DEGs and differential metabolites correlated with these metabolic pathways, and identified a total of 24 DEGs (Fig. 1A) and 23 differential metabolites (Fig. 1B). Subsequently, the authors analyzed the correlation between DEGs and differential metabolites (Fig. S4) and identified 7 key DEGs and 4 key differential metabolites. As shown in Fig. 1C, the mRNA expression levels of lipid metabolism-related genes *Acot2*, amino acid metabolism-related gene *Nos2*, carbohydrate metabolism-related genes *Itpkb*, and *Inpp4b* were significantly increased in the HFD groups compared to the NC group. However, endurance exercise significantly downregulated *Acto2, Inpp4b, Itpkb*, and *Nos2* expression compared to the HFD group. Meanwhile, compared to the NC group, HFD significantly increased the levels of lipid metabolism-related metabolites (Palmitic acid, arachidonate, phosphatidylcholine, acetylcholine), nucleotide metabolism-related metabolites (Hypoxanthine), amino acid metabolism-related metabolite (Urea), and lipid metabolism, amino acid metabolism, and carbohydrate metabolism-related metabolite (Acetyl-CoA) (Fig. 1D). Endurance exercise reduced the related metabolite levels in HFE compared to HFD groups. In addition, *Acot2* was significantly positively correlated with urea, palmitic acid, and arachidonate; *Nos2* had a positive correlation with acetyl-COA, palmitic acid, and phosphatidylcholine; *Itpkb* was positively associated with acetyl-COA, palmitic acid, and hypoxanthine; *Inpp4b* exhibited a positive correlation with acetyl-COA, palmitic acid, and phosphatidylcholine (Fig. 1E‒F).

## Discussion

In the current study, the authors investigated the effects of endurance exercise on the transcriptome and metabolome of skeletal muscle in mice fed an HFD. Integrated transcriptome and metabolome analysis revealed that endurance exercise altered lipid metabolism, amino acid metabolism, and nucleotide metabolism pathways in skeletal muscle of obese mice. Endurance exercise might have a positive impact on the metabolic health of skeletal muscle in obese mice by modulating relevant metabolic pathways and reducing the levels of specific metabolites.

Skeletal muscle serves as the primary site for the storage and metabolism of amino acids and proteins in the human body. The quality and functional integrity of skeletal muscle are essential for maintaining normal function and metabolic homeostasis within the musculoskeletal system.^12^ It has been reported that a decline in metabolism and endurance of skeletal muscle is commonly observed in obese patients.^26^ The DEGs between metabolically healthy obesity and unhealthy obesity were correlated with lipid metabolism in skeletal muscle.^27^ In the present study, a skeletal muscle transcriptome was performed to further understand the effects of endurance exercise on skeletal muscle-related metabolic processes in obese mice. GO and KEGG enrichment analyses showed that endurance exercise might reduce obesity via regulating genes involved in nucleotide metabolism and lipid metabolism in obese mice. Moreover, the small-molecule metabolic process was affected by HFD and endurance exercise in all groups. The process of small molecule metabolism involves the synthesis and degradation of many compounds in the body, including amino acids, fatty acids, and carbohydrates.^28^ A study of overweight/obese Caucasians and African Americans found differential associations of small molecule metabolites with overweight/obesity.^29^ HFD could promote the expansion of adipose tissue, disrupt lipid and sugar metabolism, create an imbalance in small molecule metabolism, and contribute to the development of metabolic diseases.^30^

Integrated transcriptomic and metabolomic analyses demonstrated that endurance exercise reduced the skeletal muscle metabolic level of palmitic acid, arachidonate, phosphatidylcholine, acetylcholine, and Acetyl-CoA in obese mice. Endurance exercise significantly reduced the expression of metabolism-related genes *Acot2* (urea, palmitic acid, and arachidonate), *Nos2*, and *Inpp4b* (acetyl-COA, palmitic acid, and phosphatidylcholine), *Itpkb* (acetyl-COA, palmitic acid, and hypoxanthine) in obese mice. Palmitic acid is a saturated fatty acid commonly found in HFD and is associated with the development of sarcopenic obesity.^31^ In cases of obesity, palmitic acid is the most abundant saturated free fatty acid.^32^ It has been shown that excess palmitic acid accumulation in adipocytes can lead to chronic low-grade inflammation^33^ and adipocyte dysregulation^34^, such as reduced secretion of adiponectin. Obesity promotes ectopic fat accumulation, skeletal muscle atrophy, and inflammatory mediators, leading to metabolic disorders in skeletal muscle.^35^ Both reduced fatty acid oxidation and increased fatty acid synthesis have been reported to increase lipid accumulation in adipocytes.^36^ Thus, after endurance exercise, decreased metabolic levels of palmitic acid in obese mice might indicate an increase in fatty acid oxidation. Research has demonstrated that endurance exercise can increase fatty acid oxidation capacity of skeletal muscle in obese mice.^37^ Through endurance exercise, skeletal muscles might utilize fat more efficiently as an energy source, thereby reducing the accumulation of palmitic acid and the associated inflammation and metabolic stress.

Arachidonate plays an important role in inflammatory responses and cell signaling.^38^ Obesity is strongly associated with chronic low-grade inflammation^39^, and endurance exercise is thought to have anti-inflammatory effects.^40^ Therefore, endurance exercise might help reduce obesity-related inflammatory responses by reducing arachidonic acid levels in skeletal muscle. Skeletal muscle atrophy is a common complication of obesity.^41^ Research showed that exercise can enhance the metabolism of acetylcholine, making its role in nerve conduction more efficient, thereby improving muscle neural control and movement ability.^42^^,^^43^ Accordingly, in mice fed an HFD, endurance exercise might affect acetylcholine levels by improving neuromuscular function and enhancing muscle contractility. Acetyl-CoA is a key intermediate in energy metabolism, and it is mainly involved in fatty acid biosynthesis and energy metabolism.^44^ In this study, the authors observed decreased acetyl-CoA levels in the skeletal muscle of exercised obese mice. However, the interpretation of this finding is multifaceted and warrants caution. The extraction method used may have compromised acetyl-CoA stability, potentially affecting its quantification.^45^ Moreover, reduced acetyl-CoA in muscle could also indicate impaired mitochondrial metabolic flux rather than a direct suppression of lipid synthesis.^46^ Therefore, future studies utilizing targeted metabolomics are essential to further explore the functional relationship between diminished acetyl-CoA availability, reduced fatty acid synthesis, and the alleviation of obesity following endurance exercise.

A key strength of this study lies in the integration of transcriptomic and metabolomic data combined with pathway-level analysis, which enabled the identification of coordinated gene-metabolite alterations and provided a holistic view of skeletal muscle metabolic adaptations to endurance exercise in the context of obesity. This multi-omics approach offers deeper biological insights than single-omics analyses alone. However, several limitations should be acknowledged. First, the small sample size (*n* = 3 per group) for omics analyses may limit statistical power and generalizability. Second, the use of an animal model, while necessary for tissue-level profiling, warrants caution when extrapolating findings to humans. Third, only male mice were included, precluding assessment of sex-specific responses. Fourth, the cross-sectional design captures molecular changes at a single time point, lacking dynamic temporal resolution. Fifth, the absence of protein-level or functional validation limits mechanistic interpretation. Finally, the untargeted nature of metabolomics may affect quantification accuracy, particularly for labile metabolites such as acetyl-CoA. Future studies incorporating larger cohorts, both sexes, longitudinal sampling, and functional validation are warranted to corroborate and extend these findings.

In summary, this study provides an integrated view of transcriptomic and metabolomic changes in the skeletal muscle of obese mice following endurance exercise. By combining these two omics datasets, the authors identified coordinated alterations in genes and metabolites within lipid metabolism, amino acid metabolism, and nucleotide metabolism pathways. Specifically, endurance exercise was associated with reduced expression of metabolism-related genes (*Acot2, Nos2, Itpkb, Inpp4b*) and decreased levels of key metabolites (palmitic acid, arachidonate, acetyl-CoA). These findings suggest that endurance exercise may contribute to improved metabolic health in obese mice through modulating multiple interconnected pathways. However, further studies with larger sample sizes and functional validation are needed to confirm these observations and elucidate the underlying mechanisms.

## Ethical approval and consent to participate

This study was carried out in accordance with the principles of the Basel Declaration (https://animalresearchtomorrow.org/en). Animal experiments were approved by the Animal Experiment Ethical Review Committee of Tianjin University of Sport's Medical Ethics Committee Tianjin (TIUS2320–010). Animal experiments were approved by the Animal Experiment Ethical Review Committee of Tianjin University of Sport's Medical Ethics Committee Tianjin (TIUS2320–010).

## Consent for publication

Not applicable.

## Data availability statement

The data supporting the conclusion are included in this article; further inquiries can be directed to the corresponding author.

## Authors' contributions

Ning Jiang participated in the design of this study, and Yu Song, Zhe Wang, Qian Wang and Huaduo Wu performed statistical analysis. Yumo Dong, Xin Gao and Yaning Song carried out the study and collected background information. Yunlong Cui drafted this manuscript. All the authors have read and approved the final manuscript.

## Funding

This study was supported by the Tianjin Education Commission Research Program Project [NO. 2021KJ002].

## Declaration of competing interest

The authors declare no conflicts of interest.

## Appendix Supplementary materials

Image, application 1

Image, application 2

Image, application 3

Image, application 4

## Footnotes

Supplementary material associated with this article can be found, in the online version, at doi:10.1016/j.clinsp.2026.101009.
