# Supplementary material for: Integrative profiling of transcriptome and metabolome of skeletal muscle after endurance exercise in a high-fat diet
Source: Clinics (Sao Paulo). 2026 May 28;81:101009. doi: 10.1016/j.clinsp.2026.101009 (PMC13235382; doi:10.1016/j.clinsp.2026.101009)

CLINICS-D-25-01912_Supplementary Material

**Table S1** The differentially expressed genes between HFD and NC groups, HFE and HFD groups, and NC and NE groups.

**Table S2** The significantly enriched GO and KEGG pathways of DEGs in HFD vs. NC, HFE vs. HFD, and NC vs. NE.

**Table S3** The significantly enriched KEGG pathways of differential metabolites in HFD vs. NC, HFE vs. HFD, and NC vs. NE.

**Figure S1 PCA plot of skeletal muscle transcriptome profiles**. HFD vs. NC (A). HFD vs. HFE (B). NC vs. NE (C).


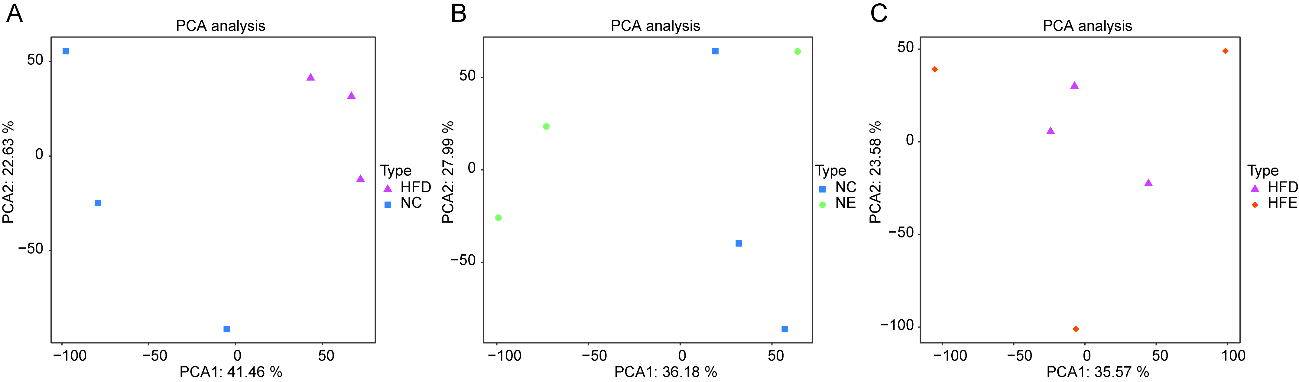


**Figure S2 Bubble diagram.** Size of dots represents genes number in each GO term.


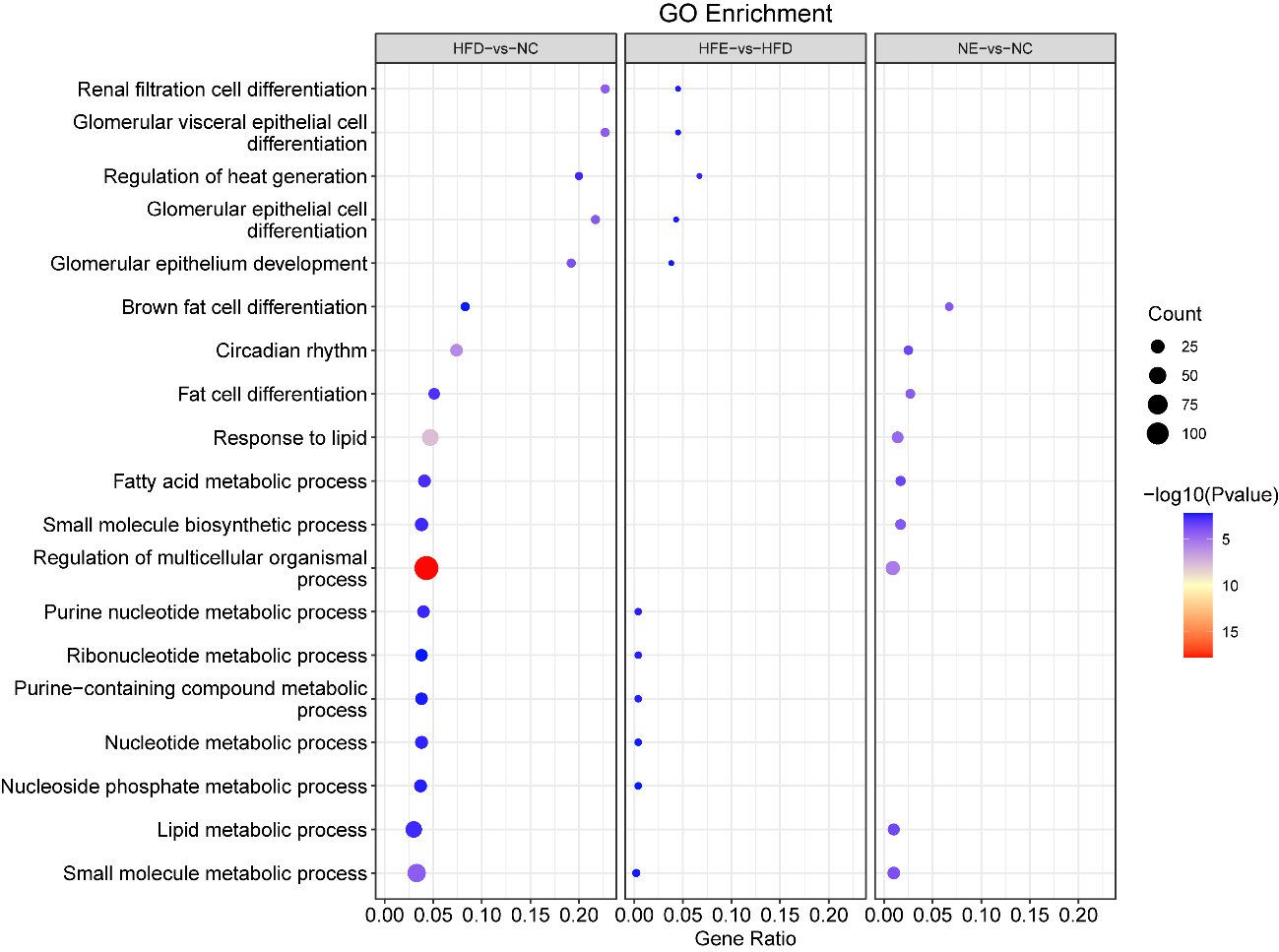


**Figure S3 S-plot of OPLS-DA model.** HFD vs. NC (A). HFD vs. HFE (B). NC vs. NE (C).


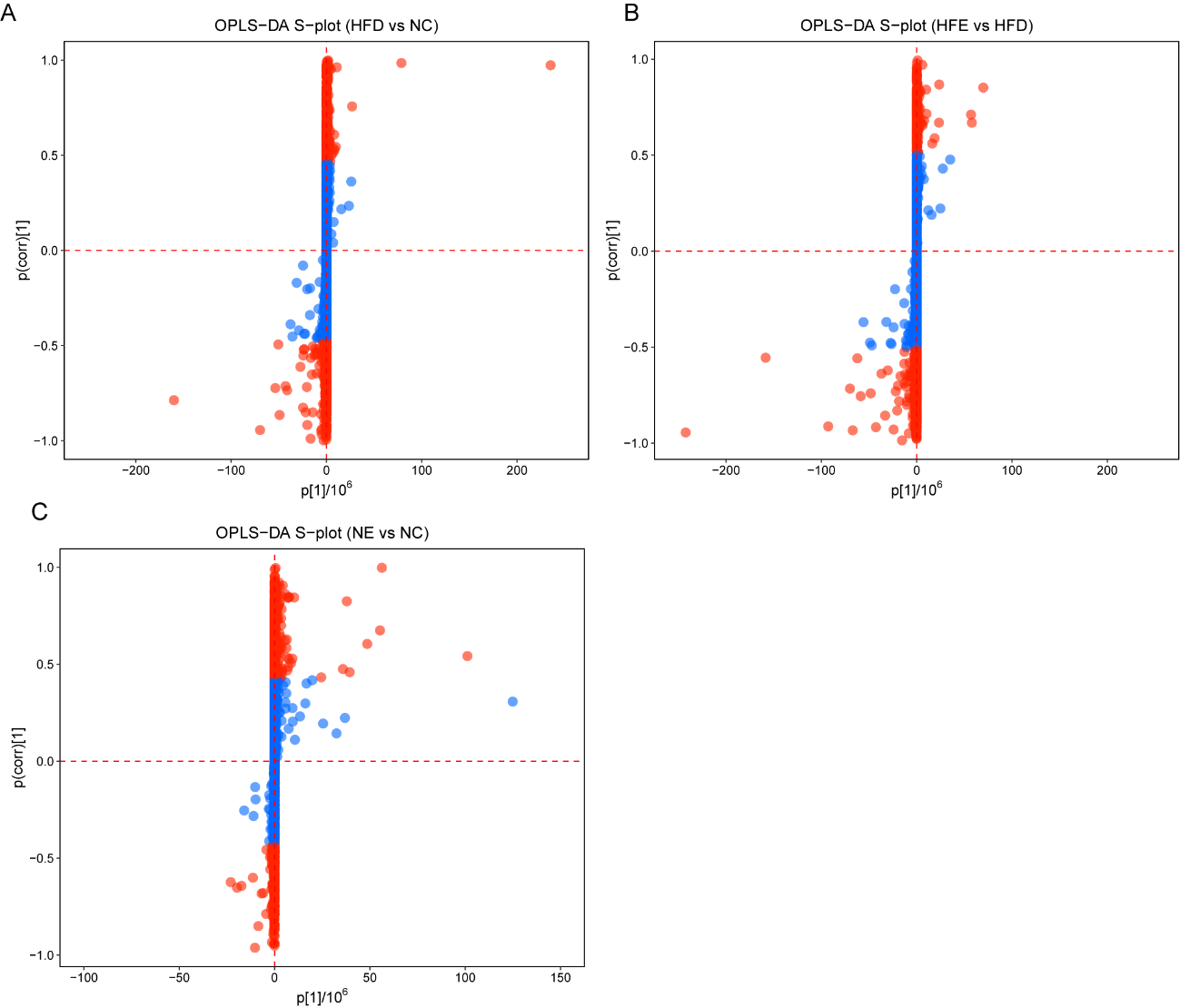


**Figure S4 Correlation heatmap of all differential genes and differential metabolites associated with key metabolic pathways.** HFD vs. NC (A).


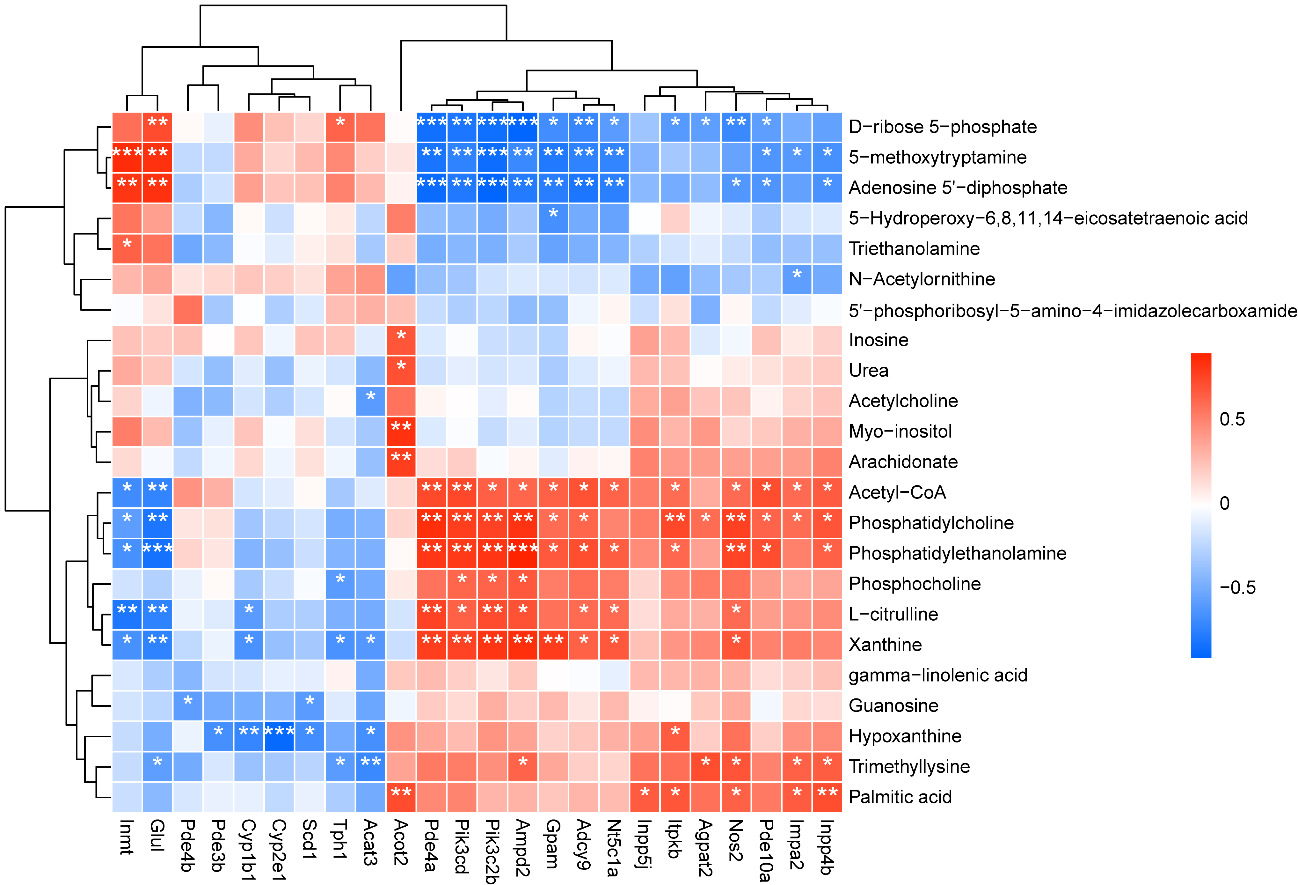

Supplement: Supplementary file 1 [file mmc1.docx]
